# Carotenoids from Marine Microalgae: A Valuable Natural Source for the Prevention of Chronic Diseases 

**DOI:** 10.3390/md13085128

**Published:** 2015-08-14

**Authors:** Maria Filomena de Jesus Raposo, Alcina Maria Miranda Bernardo de Morais, Rui Manuel Santos Costa de Morais

**Affiliations:** CBQF—Centro de Biotecnologia e Química Fina—Laboratório Associado, Escola Superior de Biotecnologia, Universidade Católica Portuguesa/Porto, Rua Arquiteto Lobão Vital, Porto 4202-401, Portugal; E-Mails: fraposo@porto.ucp.pt (M.F.J.R.); abmorais@porto.ucp.pt (A.M.M.B.M.)

**Keywords:** carotenoids, marine microalga, astaxanthin, β-carotene, fucoxanthin, ROS, RNS, free radicals, oxidative stress, inflammatory diseases, chronic diseases, aging

## Abstract

Epidemiological studies have shown a relation between antioxidants and the prevention of several chronic diseases. Microalgae are a potential novel source of bioactive molecules, including a wide range of different carotenoids that can be used as nutraceuticals, food supplements and novel food products. The objective of this review is (i) to update the research that has been carried out on the most known carotenoids produced by marine microalgae, including reporting on their high potentialities to produce other less known important compounds; (ii) to compile the work that has been done in order to establish some relationship between carotenoids and oxidative protection and treatment; (iii) to summarize the association of oxidative stress and the various reactive species including free radicals with several human diseases; and (iv) to provide evidence of the potential of carotenoids from marine microalgae to be used as therapeutics to treat or prevent these oxidative stress-related diseases.

## 1. Introduction

Changes in lifestyle and nutrition, stress most people are submitted to, growing levels of pollution, exposure to xenobiotics and radiation that has been increasing for the last decades, and even an excess of physical exercise may all be responsible for several metabolic diseases. These factors can cause changes in the metabolism, increasing the cellular respiration rate and inducing an imbalance between the oxidation products generated and the natural antioxidant capacity of the cells. During the normal metabolism, mainly along the respiratory chain in the mitochondria, some free radicals and reactive species, either from oxygen (ROS) or nitrogen (RNS), but also proteins and lipids, are naturally elicited to suffer (per)oxidation. DNA can be a target as well, leading to genome instability and increasing the risk of molecular change and mutations. All these changes may cause damage in the cell metabolism, disease and even death. It is known that ROS are associated with a wide range of diseases, such as aging, Alzheimer, Parkinson, atherosclerosis, cardiovascular disease (CVD), cancer, inflammatory and neurological diseases, diabetes and obesity, amongst others [1,2,3]. RNS are also involved in the peroxidation of lipids and nitration of proteins, as well as in the formation of many other reactive species, such as malondialdehyde (MDA), a marker of oxidative stress.

As with mitochondria, cell organelles that are very vulnerable to oxidative stress due to the high concentration of oxygen (O_2_), the brain and the central nervous system (CNS) are also susceptible to lipid/fatty acid peroxidation induced by ROS, because of the high pressures of O_2_ and oxidation reactions. This may be the main reason why neurodegenerative diseases—Alzheimer, Parkinson, amyotrophic lateral sclerosis, amongst others—are strictly associated with oxidative damage and/or deficiency of endogenous oxidative protection in the cells of the nervous system [4]. Using a simple but clarifying scheme, Kalam *et al.* [4] have synthesized the whole process starting with the formation of free radicals, ROS and RNS, and continuing on through the protective responses of antioxidants (AO) and the effects of oxidative stress on various macromolecules, which lead to a wide range of chronic diseases. Another organ susceptible to being attacked by ROS are the lungs, as they are also exposed to a high concentration of O_2_ and radicals and other reactive species originating from pollutants like cigarette smoke, such as O_2_^•^^−^ and NO, which trigger oxidative chain reactions [5].

However, some of the free radicals and reactive species that result from the cellular metabolism, are effective/beneficial in moderate concentrations in regulating the intracellular redox signaling [3], fighting the invasion of organisms and inflammation by pathogens [6], or even inducing the cells to adapt themselves and protect from other severe oxidative damages. For example, they may create tolerance to ischemic-induced conditions [7]. When this equilibrium is broken and reactive species surpass the capacity of endogenously-induced anti-oxidative protection of cells, the imbalance induces damage by oxidative stress, and regular physiological functions of proteins, lipids, DNA or other important biocompounds become impaired, triggering the pathogenesis of several diseases.

Carotenoids in general, and those from marine microalgae in particular, are excellent antioxidants, which can be exogenously supplied to the cells, re-establishing the levels of oxidative stress (OS) and nitrogen stress (NS) by neutralizing the excess of free radicals and reactive species. Some of the most known and studied carotenoids produced by microalgae include β-carotene from *Dunaliella salina*, astaxanthin from *Haematococcus pluvialis*, canthaxanthin from *Coelastrella striolata*, but also the less known, but not less effective, fucoxanthin from several diatoms, such as *Phaeodactylum tricornutum*, and *Isochrysis galbana* (Table 1).

Several studies have already been published reporting the association of carotenoids-antioxidant properties with protection against several malfunctions and diseases triggered by oxidative stress [8,9,10,11,12,13,14,15], but only a few references have dealt with carotenoids from marine microalgae [6,16,17,18,19,20,21], and even fewer studies relate to the beneficial effects of microalgal carotenoids in humans [22,23,24,25,26].

This review will focus on the microalgae producing the various carotenoids, their cumulative concentrations and the known biosynthesis pathways of carotenoids in microalgae. The antioxidant capacity of the different carotenoids against the various radicals and reactive species will also be discussed, as well as the benefits of using marine carotenoids (or microalgae biomass) to neutralize the oxidative stress, as this may trigger several diseases.

## 2. Carotenoids from Marine Microalgae

Several kinds of carotenoids are produced by marine microalgae (Table 1). Most of them have been clearly quantified, but sometimes only the percentage of the total carotenoid yield is known. Many other carotenoids have been reported to be constituents of a given microalga, but there in these cases, no reference is made regarding their concentration [27,28]. 

According to their structure, carotenoids can be classified into different groups. Carotenes (α- and β-carotenes (*D. salina*), and lycopene) do not have any substituent (or even oxygen) in their structure, they are strict hydrocarbon carotenoids; xanthophylls or oxycarotenoids present –OH groups (hydroxycarotenoids: zeaxanthin from *P. cruentum*, lutein from *C. pyrenoidosa*), =O groups (ketocarotenoids: canthaxanthin from *C. striolata*), echinenone from *B. braunii*, *S. platensis*), or both –OH and =O groups (astaxanthin from *H. pluvialis*) (Table 1). In addition, some other carotenoids have more complex structures, such as violaxanthin (*C. ellipsodea*) and diadinoxanthin (*P. tricornutum*) with epoxy groups, and dinoxanthin and fucoxanthin (*I. galbana*) with acetylated groups. These last two acetylated carotenoids, which can be found in diatoms, haptophytes and dinophytes [29], present the curious structure C=C=C (allene), unique to natural products [30]. A more curious structure, acetylene or C≡C, is also part of some carotenoids (all-, diato-, diadino-, hetero-, croco-, pyro- and monadoxanthin); these acetylenic carotenoids can be typically found in some groups of microalgae, such as the cryptophytes, diatoms, haptophytes, euglenophytes and dinophytes [29]. Some new carotenoids have been identified in *B. braunii* (Table 1).

Another curiosity is the fact that only two carotenoids have been found in the red marine microalga *P. cruentum*: zeaxanthin (making up 97.4% of the total carotenoids) and β-carotene (with only 2.6%) (Table 1).

**Table 1:** Main carotenoids from marine microalgae.

Main Carotenoid	Chemical IUPAC Name/Chemical Structure	Concentration	Microalga	Other Carotenoids	Remarks	References
β-carotene	β,β-carotene	10%–13% DW	*Dunaliella salina*	zeaxanthin, lutein, α-carotene	occur mostly as a mixture of 9-*cis* and all*-trans*, but also other *cis* isomers	[31,32,33,34,35,36,37]
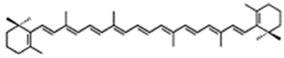		50% TC (TC = 0.9% DW)	*Chlorella zofingiensis*	canthaxanthin (25% TC or 97% DW), astaxanthin (0.7% DW)		
		80% TC	*Arthrospira*	astaxanthin, lutein β-cryptoxanthin, zeaxanthin, echinenone, oscillaxanthin, myxoxanthophyll		
astaxanthin(as 3*S*,3′*S* isomer)	3,3′-dihydroxy-β,β-carotene-4,4′-dione	up to 7% DW; 75% TC	*Haematococcus pluvialis*	β-carotene, lutein, canthaxanthin, neoxanthin, violaxanthin, zeaxanthin, echinenone	occur as a racemic mixture of mainly mono- and diesters (*ca*. 73% TC), but also as freeastaxanthin	[21,38,39,40,41,42]
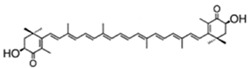						
lutein	β,ε-carotene-3,3′-diol	0.2%–0.4% DW	*C. pyrenoidosa*	violaxanthin, loroxanthin, α- and β-carotene		[43]
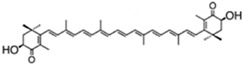						
canthaxanthin	β,β-carotene-4,4′-dione	4.75% DW	*Coelastrella striolata* var. *multistriata*	astaxanthin 0.15% DW, β-carotene 0.7% DW		[37]
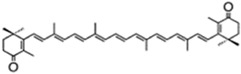						
canthaxanthin lutein	β,β-carotene-4,4′-dione β,ε-carotene-3,3′-diol	45% TC	*C. vulgaris*	astaxanthin 12.5% TCviolaxanthin		[44,45]
fucoxanthin	acetic acid [(1*S*,3*R*)-3-hydroxy-4-[(3*E*,5*E*,7*E*,9*E*,11*E*,13*E*,15*E*)-18-[(1*S*,4*S*,6*R*)-4-hydroxy-2,2,6-trimethyl-7-oxabicyclo[4.1.0]heptan-1-yl]-3,7,12,16-tetramethyl-17-oxooctadeca-1,3,5,7,9,11,13,15-octaenylidene]-3,5,5-trimethylcyclohexyl] ester	1.65%DW	*P. tricornutum*	diadinoxanthin, eaxanthin, neoxanthin, violaxanthin, β-carotene	occur mainly as all-*trans* but also as *cis* isomers	[46,47,48]
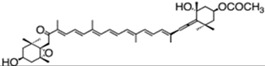		1.8% DW	*Isochrysis* aff. *galbana*			[49]
		0.52% DW	*Cylindrotheca closterium*			[50]
		up to 2.2% DW	*Odontella aurita*	diadinoxanthin, β-carotene		[51]
zeaxanthin	β,β-carotene-4,4′-diol	97.4% TC	*P. cruentum*	β-carotene		[52]
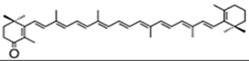						
echinenone (extracelular)	β,β-carotene-4-one	0.17% DW	*B. braunii*	botryoxanthins A and B—0.03% DW braunixanthins 1 and 2—0.06% DW	extracellular pigments are produced and secreted into the intercellular matrix	[53]
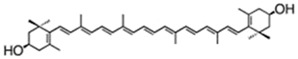						
Lutein (intracelular)	β,ε-carotene-3,3′-diol	up to 0.16% DW		neox/loroxanthin 0.042% DW α- and β-carotene 0.031% DW violaxanthin 0.02% DW		
violaxanthin	(1*S*,4*S*,6*R*)-1-[(1*E*,3*E*,5*E*,7*E*,9*E*,11*E*,13*E*,15*E*,17*E*)-18-[(1*S*,4*S*,6*R*)-4-hydroxy-2,2,6-trimethyl-7-oxabicyclo[4.1.0]heptan-1-yl]-3,7,12,16-tetramethyloctadeca-1,3,5,7,9,11,13,15,17-nonaenyl]-2,2,6-trimethyl-7-oxabicyclo[4.1.0] heptan-4-ol		*C. ellipsodea*	antheraxanthin, zeaxanthin		[45]
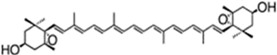						

TC = total carotenoids.

**Figure 1:**
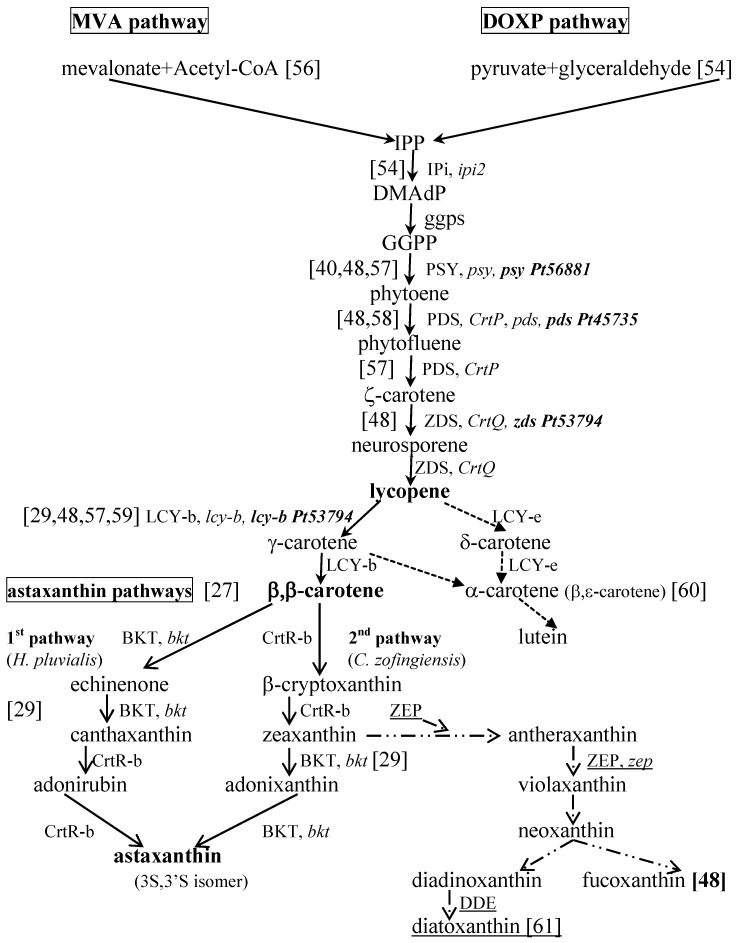
Pathways for the carotenogenesis and biosynthesis of astaxanthin, based on Han *et al.* [27], Dambek *et al.* [48] and Takaichi [29], and the synthesis of fucoxanthin and diadinoxanthin in diatoms [61], including *P. tricornutum*, proposed by Dambek and colleagues [48]. The genes and enzymes involved in the process and already identified in some microalgae are evidenced; the underlined ones were reported by Bertrand [61].

The biosynthesis of isopentenyl pyrophosphate (IPP) may usually be explained by one of two different pathways (Figure 1): the mevalonate pathway (MVA) in euglenophytes and 1-deoxy-d-xylulose-5-phosphate pathway (DOXP) in Chlorophyceae and Cyanophyceae [54,55].

Based on Takaichi [29] and Han *et al.* [27], Figure 1 summarizes the carotenogenesis pathways for eukaryotic microalgae, assuming the DOXP pathway for IPP synthesis (Figure 1) and the scheme indicated by Takaichi [29] for the pathways in cyanobacteria. Some known genes and the enzymes they code for have been isolated from different species of microalgae and cyanobacteria, and their functions have been conveniently identified and presented in recent reviews [27,29].

Concerning the derivatives of either β- and α-carotene, most of the steps indicated for the full process of the carotenogenesis pathway in algae have been proposed by establishing some relations among the chemical structures of carotenoids [29]. However, the real pathways and enzymes involved still need to be studied and explained.

## 3. Oxidative Stress—The Importance of Reactive Species in Aging and Age-Related Diseases

**Figure 2:**
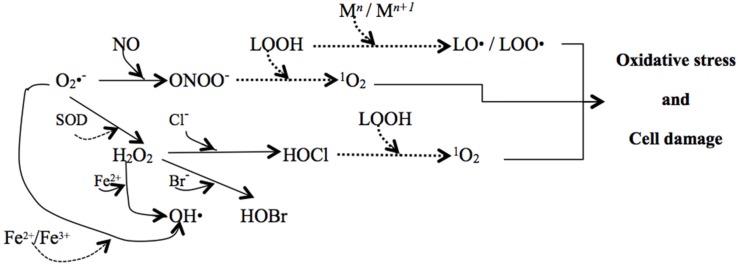
Cascade triggered within a cell by O_2_^•−^ (superoxide radical) generated mainly by NADPH oxidase. Some reactions with lipid hydroperoxides (LOOH) are also included (dashed arrows; M = transition metal) [58,63,64,65].

Once initiated, the propagation of (per)oxidative reactions, spread out by the reaction of the new formed products or free radicals with other chemical species, including peroxyl and alkoxyl radicals, which in turn can go on attacking lipids and other biomolecules. For example, peroxynitrite, the product of the reaction between superoxide anion radicals and nitric oxide can generate, in the presence of lipid hydroperoxides, an oxygen singlet (Figure 2), which is another reactive species (not a free radical) able to cause severe modifications in amino acids and/or proteins [62].

Free radicals are generated during cell metabolism, and their levels may be increased by pro-oxidants. They need to receive an electron to pair with the unpaired electron. When these important chemical reactive species are overproduced *in vivo* they can cause an imbalance between ROS and RNS and their elimination by the endogenous AO, including enzymes (catalase, for example) and glutathione (GSH), which are part of the cell defense mechanisms [4]. The oxidative stress generated will induce the chain oxidation reactions, causing cell damage and aging, and, therefore, inducing several diseases, including cancer [66,67]. 

However, ROS and RNS have addition beneficial effects that are strictly related to cell signaling and the immune system, *i.e.,* to supply energy and to get rid of toxins [68], a major role they play when they are present in small amounts. When this redox equilibrium is broken, ROS can attack lipids, proteins and DNA, forming lipid radicals, amino and thiyl radicals, and sugar- and base-derived radicals that, in turn, can be oxidized into peroxyl radicals (Table 2) [4]. Kalam *et al.* [4] have simply and clearly described the (per)oxidation chain reactions starting with the attack of ROS on various essential biomolecules, especially lipids, continuing on to the formation of lipid hydroperoxydes and peroxyradicals, and subsequently generating aldehydes. Some of the newly formed radicals can be scavenged by the endogenous AO, but others can feed the oxidative chain reaction. 

**Table 2:** Reactive species causing oxidative stress.

Groups of Reactive Species	Examples
ROS	O_2_^•^^−^, H_2_O_2_, HO^•^, ^1^O_2_, O_3_
RNS	NO^•^, ONOO^•^, NO_2_^−^
Lipid hydroperoxides	LO^•^, LOO^•^
RCS	HOCl, HOBr
Glycoxidative species	AGE, ALE
Others:	Carbonyl radicals, RSS, GS^•^

RCS = reactive chlorous/bromous species; RSS = reactive sulfur species; GS^•^ = glutathione thiyl radical.

Moreover, the reactive aldehydes, such as MDA, hydroxynonenal (4-HNE) and acrolein (ACR), and lipid hydroperoxides seem to play a concentration-dependent dual role in the cell metabolism [62]. On the one hand, they can show protective functions by acting as signaling molecules and stimulating gene expression or neutralizing peroxidative damages by reducing themselves while receiving two electrons [62]. On the other hand, as they are highly reactive and toxic, these reactive species may be reduced by receiving one electron, initiating the lipid peroxidation cascade [69] and causing considerable damage in the cells, sometimes even leading to death [62]. These reactive species can also inhibit gene expression, and they play an important role in neurodegenerative diseases. This dual role depends on the cellular levels and cell types of the reactive aldehydes: They may present some oxidative protection in spite of eventually inducing some cell damage with which cells can survive and subsist, or they may cause severe effects leading to the development of several diseases [62]. Compared to free radicals, these aldehydes are relatively stable and can be more dangerous than ROS as they can exert their action away from the place where they were formed [70], whilst oxygen radicals are short-live species that attack in the surroundings of the site of production [71]. Nevertheless, these can also be dangerous. In fact, HO^•^, which is able to start lipid peroxidation close to the site of origin, generating LOO^•^ radicals, is a very reactive species [6]. 

Ayala and colleagues have recently [62] and extensively given an overview of the relationship between oxidative stress and disease; they present simple but clarifying schemes to elucidate the mechanisms of lipid peroxidation, especially MDA and 4-HNE formation and metabolism.

Oxidative stress has also been associated with the formation of atheromas that can lead to cardiovascular diseases [3,72,73], including thrombosis after rupture of atherosclerotic plaques. Overproduction of free radicals is another feature at the origin of pathological inflammation [3]. By using simple schemes, Pashkow [3] has explained the onset of CVD, starting with oxidative stress, which greatly interferes with the regular function of platelets. These cell particles release more NO, a reactive element that induces an increase of the thrombus due to platelets aggregation. A treatment with a ROS scavenger has been suggested to inhibit such platelet behavior, thus stimulating their disaggregation. Furthermore, diabetic cardiomyopathy, congestive cardiomyopathy and hypertension-related heart disease were some of the diabetic complications arising from oxidative stress that were given in the overview by Pashkow [3].

Klaunig *et al.* [74] are another group that worked on the link between oxidative damage and disease, namely carcinogenesis. They suggested that the increase in number and size of peroxisomes (cell organelles), which are induced by hypolipidemic drugs and other substances, may be associated to liver cancer [75]. This may be explained by an increase of the peroxisomal enzymes acyl-CoA and xanthine oxidase (XO) induced by oxidative stress that enhances the production of ROS, such as H_2_O_2_ and O_2_^•^^−^.

Another reason for the association of oxidative stress with disease is the fact that F2 Isoprostanes (e.g., 8-iso-PGF_2_α) are biomarkers associated with lipid peroxidation. They are indicators of the presence of diabetes 1 and 2 in the urine [76]. The formation of these substances and the subsequent increase of thiobarbituric acid reactive species (TBARS) (Table 3) can be inhibited by the endogenous superoxide dismutase (SOD) and butylated hydroxy-toluene (BHT) [77]. Perhaps, a similar response might be obtained by using carotenoids, as they, or at least some of them, are able to neutralize oxygen free radicals (Table 4). Moreover, oxidative stress has been associated with hyperglycemia for decades [73,78,79]. Mezzetti and colleagues [76] have concisely established various links between the different sources of oxidative stress and the associated biomarkers (Table 3), which also appear in higher levels in plasma and/or urine of diabetics and patients with related complications, such as atherosclerosis.

Additionally, in diabetics for example, the autoxidation of sugars, plasma unsaturated lipids and proteins from cell membranes, and the reactions between sugars and proteins produce free radicals, which may be the cause of oxidative stress and damage. Baynes [8] established some relationships between oxidative stress and the subsequent effects/damages in diabetic patients. He described some sources of oxidative stress, and suggested some therapeutical treatments using antioxidants and anti-inflammatory agents, other than carotenoids, to reduce the effects of oxidized lipoproteins and lipid peroxides. These relationships may explain the (per)oxidative and inflammatory processes as triggering the initial steps of diabetes, but also complications derived from the development of the disease itself. This type of therapeutics can be used to break down the chain-production of free radicals [8] as well. Baynes [8] also reviewed the reactions that may lead to atherosclerosis, as a complication generated by the oxidation of lipids and lipoproteins in diabetics. Some biomarkers for oxidative stress (Table 3) in these patients were given as well, as some of those glycative and/oxidative products (Table 3) are unique and stable.

In addition, one of the theories on aging claims that it might result from damaged tissues caused by reactive species, such as free radicals that are generated during the consumption of oxygen by mitochondria. During aerobic respiration, the agents responsible for the AO defense are overwhelmed by these ROS and an imbalance is created. By using the right natural carotenoids, including those from marine microalgae, at the corrected doses, the levels of such reducing agents would be expected to improve, restoring the endogenous antioxidative system in the cells [80].

Furthermore, according to Kalam and co-workers [4], it seems that the initiation process in chronic diseases is always associated with the overproduction of reactive species (e.g., ROS), which mediate the initial steps of inflammation that leads to carcinogenesis, directly by the oxidation, nitration or halogenation of lipids or nucleic acids, or indirectly by activating the signaling pathways.

**Table 3:** Main biomarkers of oxidative stress.

Biomarkers	Remarks	References
MDA	reactive aldehyde; lipid peroxidation of ω-3 and ω-6 FA	[81]
4-HNE	reactive aldehyde; lipid peroxidation of ω-3 and ω-6 FA	[81]
acrolein	reactive carbonyl	[5]
LOOH	lipid hydroperoxides	[82]
Glycoxidation products:	carbonyl-derived from protein changes	[8,76,83]
pentosidine (AGE)	marker for diabetes-associated complications	
*N*^ε^-(carboxymethyl)lysine (AGE)	(CML); marker for diabetes-associated complications	
*N*^ε^-(carboxymethyl)hydroxylysine	(CMhL) (AGE)	
*o*-, *m*-, and dityrosine		
fructoselysine	marker for diabetes	
fructosehydroxylysine		
ketoimine and ketoamine adducts	to protein	
ALE	advanced lipoxidation end products	
Dopaminergic markers: tyrosine hydrolase dopamine transporter		[84,85]
Cell apoptosis markers (hallmarkers): DNA fragmentation condensation of cell nuclei		[86]
PARP breaking	poly ADP ribose polymerase	
Markers for PD’s substantia nigra: 8-OHdG MDA LOOH protein carbonyls		[74]
Markers for oxidative stress in tissues: 8-OHdG* 8-nitroguanine* 4-HNE		[22,73,74,76,78,79,87,88,89,90,91]
GSH	Glutathione	
isoprostanes (e.g., IPF2α-I)	useful for atherosclerosis plaques; markers for lipid peroxidation *in vivo*	
LOOH & thiobarbituric acid reactive species (TBARS)		
enediol radical anion	marker for diabetic patients; results from autoxidation of glucose	

* useful for DNA lesions and human cancers.

## 4. Carotenoids from Marine Microalgae against Oxidative Stress

### 4.1. Uptake and Bioavailability

Most carotenoids cross the cell membrane by simple or facilitated diffusion [92,93,94,95] and are delivered through the body as a part of lipoproteins [93,94,95,96,97]. All-trans and 9-*cis* forms of β-carotene are mainly accumulated in chylomicrons and VLDL, respectively [98], and xanthophylls in LDL and HDL [99,100]. However, astaxanthin appears mostly in VLDL-containing chylomicrons [101], and lutein and zeaxanthin are found to be preferably absorbed into chylomicrons [102].

According to several groups of researchers, carotenoids are better available if administered along with some kinds of fat, such as phospholipids or special edible oils [26,92,103,104] as the vehicle, since carotenoids are better absorbed into chylomicrons at the intestine level. From here they are delivered to the cells of the individuals/animal models, where they seem to be especially integrated into the mitochondria [86,96,105], but also into nuclei and microsomes [96]. However, different carotenoids seem to be absorbed differently. For example, fucoxanthin has been found to being better absorbed than lutein or astaxanthin, the metabolites of these being found in higher concentrations in mice [106,107]. Furthermore, these metabolites, fucoxanthinol, amarouciaxanthin A and halocynthiaxanthin, have already been identified in several animal models and cell-lines [108,109,110,111]. Some studies have even suggested a better bioavailability of fucoxanthinol than fucoxanthin [112], and have reported that the former is converted into amarouciaxanthin A in the liver [103]. Additionally, no fucoxanthin has been found in the liver and plasma [110,113], but fucoxanthinol and amarouciaxanthin have been detected in several tissues [107]. More recently, Rao and co-workers [114] found that astaxanthin, β-carotene and lutein from different microalgae appeared mainly in the liver, with astaxanthin being preferentially absorbed; this carotenoid also showed a higher AO capacity by increasing catalase, SOD and peroxidase to higher levels and by preventing *in vivo* lipid peroxidation. Additionally, astaxanthin demonstrated to be better available and accumulated in larger amounts than lutein and β-carotene in a rat-model. It is worth noting though that in this study [114], the results among different groups of animals could have been influenced by other compounds of the microalgal biomass, since the various carotenoids were administered as being part of referred biomass from *H. pluvialis*, *S. platensis* and *B. braunii.* In addition, natural carotenoids already have been shown to be better available than synthetic ones [115], and the availability is also improved if carotenoids are taken after meals [116]. Furthermore, Régnier *et al.* [21] found that a natural racemic mixture of astaxanthin extracted with solvents presented twice as much antioxidative protection as that extracted by supercritical methods; this protection was almost five times higher (*in vitro* tests) than that of synthetic astaxanthin, which presented even lower activity than astaxanthin obtained by supercritical methods. However, when AO activity of various extracts were tested in Human Umbilical Vein Endothelial Cells (HUVEC) cells, the antioxidative cell protection was almost 90 times higher with the natural astaxanthin containing esters than with the synthetic xanthophyll [21], thus contributing to the cells’ defense against oxidative stress. Regarding astaxanthin, the geometrical isomer 3*S*,3′*S* was more readily absorbed despite being in a lower concentration in a racemic mixture, and *cis* (*Z*) forms were selectively absorbed [117], especially 13*Z* isomers [101].

### 4.2. Antioxidant Protection

In the near future, unicellular microalgae might be an alternative source to obtain AO because their carotenoids (Table 4), phenolics and other bioactive compounds, such as vitamins, fatty acids and polysaccharides, act synergistically towards their AO properties, which may be of some usefulness against oxidative damage [118].

**Table 4:** Antioxidant activity of main carotenoids from microalgae against reactive species.

Carotenoid	AO activity	Reactive Species	References
astaxanthin	^1^O_2_ quencher,	^1^O_2_,	[6,86,119,120,121,122,123]
	radical scavenging,	O_2_^•^^−^, H_2_O_2_, HO^•^,	
	ROS and RNS quencher,	NO, LOOH, ONOO^−^, HOCl	
	chain-breaking AO, lipid peroxidation inhibitor, inhibits hallmarkers		
β-carotene	^1^O_2_ quencher;	^1^O_2_,	[6,124,125,126]
	radical scavenger;	NO_2_, ONOOH and ONOO^−^	
	inhibits Na^+^K^+^-ATPase, stimulates catalase and GS transferase		
canthaxanthin	ROS and RNS quencher; chain-breaking AO	^1^O_2_	[119,121]
fucoxanthin	^1^O_2_ quencher,	^1^O_2_,	[6,103,124,127,128,129,130,131,132,133]
	radical scavenger;	O_2_^•^^−^, HO^•^,ONOO^−^, HOCl,	
	inhibits Na^+^K^+^-ATPase, stimulates catalase and glutathione transferase	DPPH^•^, 12-DS^•^, NB^•^-L, AAPH, ABTS, ABAP, AIBN	

There is evidence that a diet rich in carotenoids and other bioactive compounds protects from several diseases, including obesity and cancer; oxidative stress is known to be associated with those diseases and malfunctions; hence, carotenoids might inhibit the onset of these diseases due to their AO activity. It is also known that carotenoids exhibit their antioxidative properties by being good scavengers of several chemical radicals and potent quenchers of singlet oxygen (Table 4).

In order to deactivate reactive species and stop oxidative reactions, AO (vitamin C, thiol, fucoxanthin, polyphenols) usually donate electrons, but some can also be proton donors, as happens with fucoxanthin, GSH and α-tocopherol [132]. Some of them act as “real” cellular AO (GSH), some others are chain-breaking AO as they can stop oxidative reactions, such as α-tocopherol [134].

The unique molecular structures of some carotenoids, which have additional oxygenic functional groups (epoxy, hydroxyl, carbonyl and/or carboxyl), together (or not) with special unique allenic features (Table 1) and several conjugated double bonds, provide them with a strong AO activity, as happens with fucoxanthin [124]. Algal fucoxanthin has already demonstrated to effectively protect from ROS and oxidative-induced damages [135], with the AO activity being higher than that of α-tocopherol. Rodrigues and colleagues [6] also found that fucoxanthin was one of the strongest carotenoids scavenging HOCl, only surpassed by astaxanthin. However, in another study, β-carotene showed to be better a ^1^O_2_ quencher [124], and halocyanthiaxanthin was the best suppressor of O_2_^•^^−^ among about twenty carotenoids, despite demonstrating the lowest scavenging properties [136]. In contrast, Pashkow and colleagues [137] describe that astaxanthin has the highest AO capacity among several carotenoids, including β-carotene, zeaxanthin and canthaxanthin, with it being 500-fold higher than that of vitamin E as a ^1^O_2_ quencher [138], and 100 times greater than that of α-tocopherol with respect to the protective effects against lipid peroxidation [139]. The AO activity of astaxanthin as a scavenger of various reactive species (LOO^•^, HOCl and ONOO^−^) was also demonstrated by Rodrigues’ group [6]. It was even more potent than α-tocopherol. Regarding RNS. However, scarce information is available concerning the capacity of carotenoids to scavenge other radicals, such as ONOO^−^. However, Rodrigues and co-workers [6] found that β-carotene was the best ONOO^−^ scavenger among several carotenoids. Also, carotenoids with oxygenic substituents, especially –OH, which can easily donate protons, showed to be better than the hydrophobic carotenes at neutralizing ROS. This dependency on the amounts of oxygen of carotenoids influencing the sensitivity to radicals and their AO was observed by other researchers as well [132,138], with the AO activity usually being inversely correlated with oxygen tension [125].

Despite the lower concentration in carotenoids, mainly astaxanthin, their extraction from palmeloid cells of *H. pluvialis* is easier and their content is more readily available. This is a positive aspect when whole cells are administered to produce antioxidative properties of their carotenoids. Owing to the absence of a hard cell wall, these reddish palmeloid cells are more suitable for human consumption [42]. In certain conditions, when the content in astaxanthin is lower, the fatty acid (FA) content may also play a role in the AO capacity [118] of *H. pluvialis*, perhaps because astaxanthin is present mainly as mono- and diesters, especially with oleic acid. According to Cerón *et al.* [42], the AO power of astaxanthin *in vitro* against the radical DPPH is 10 times higher than that of β-carotene, 500 times higher than that of α-tocopherol, and diesters are 1.6 times more potent than monoesters and twice as potent as free astaxanthin. In addition to the AO potential, astaxanthin is also an antiglycant [28]. Therefore, this carotenoid is able to protect from glycation of proteins, which may be subsequently oxidized to form glycoxidation products (Table 3). However, protein oxidation products can have a positive oxidative stress response as well. S-nitrosylation of a single cysteine residue and S-sulphenation of cysteine, where sulphydryl group of cysteine is converted to a S-sulphenation product, or addition of a group –SOH to DJ-1 protein is related to Parkinson’s disease; this oxidation product has a positive effect and is neuron protective [7].

Additional protection in cells may come from the beneficial activity of some ROS that can interact with NO to form peroxynitrite (Figure 2), which in turn can react with carotenoids to form nitrocarotenoids that scavenge those radicals. However, few reports exist on this matter [123,129]. Peroxynitrite, a reactive nitrogen species, is responsible for the nitration of tyrosine, as a free aminoacid or as a monomer in proteins, and for the oxidation of LDL, peroxidation of lipids and fragmentation of DNA [140]. Fucoxanthin inhibits nitration of tyrosine by reacting with peroxynitrine and forming *Z-*nitrofucoxanthin [129], thus, proving the capacity to neutralize those radicals. These new products also showed to have anti-tumor and anti-proliferative properties, which are induced/triggered by RNS. This anti-carcinogenic activity of nitrofucoxanthin has even been found to be higher than that of fucoxanthin [129]. Astaxanthin and lutein can also scavenge RNS ONOO^•^ to form 15*Z*-nitroastaxanthin and 15*Z*-nitrolutein that, besides maintaining antioxidative activity against oxygen singlet and inhibiting nitration of tyrosine, has shown (as it happens with nitrofucoxanthin [129]) anti-tumorigenic-induced RNS properties higher than those of astaxanthin and lutein [123]. However, astaxanthin demonstrated an AO activity as a ^1^O_2_ quencher higher than nitroastaxanthin, and even higher than β-carotene; lutein and nitrolutein have similar ^1^O_2_ quenching activities, higher than β-carotene [123]. Astaxanthin showed no cytotoxicity for several kinds of cell-lines, either healthy or tumor cells, when in doses up to 16.75 μM [21]. Additionally, no side effects were noticed in the studies reported and reviewed by Fassett and Coombes [13].

In what concerns fucoxanthin, in addition to its high AO activity, this unique carotenoid is also able to modulate certain genes involved in the cell metabolism [141], and these properties seem to be essential for health. However, fucoxanthin metabolites, fucoxanthinol and amarouciaxanthin A, seem to be responsible for the physiological effects in the gastrointestinal tract and liver, respectively, in mice and rats [107,109,142], and the conversion from fucoxanthin into its metabolites has been observed in Caco-2 and HepG2 human cell-lines as well [92,109,110]. Nevertheless, as drugs in general have a shorter retention time in smaller animals [143], the effects of bioactive compounds, such as fucoxanthin and the respective metabolites, cannot be directly compared with the correspondent in humans, where bioavailability is higher due to a slower metabolism. In addition, absorption of fucoxanthin, and perhaps other carotenoids, depends on the food ingested, since its solubility is low in vitamin E and some vegetable oils, but is rather dissolved in medium-sized triacylglycerols and fish oil [144,145]. Furthermore, as it happens with β-carotene, the chemically synthesized fucoxanthin might not demonstrate the same functionalities as those from the natural racemic *cis*-*trans* mixtures. Regarding safety, fucoxanthin has proven to be a safe product in several studies with different cell-lines and animal models, either administered in one single dose or given over a period of time [146,147], as reported by Peng *et al.* [103]. Fucoxanthin from marine algae has also proven to be able to regulate lipidemic marker levels in the blood and liver of various (obese) animal models and reduce their white adipose tissue and body weight as well [148,149,150,151,152,153]. A decrease of body weight, body and liver fat in obese women treated with Xanthigen, some of them with non-alcoholic fatty liver disease (NAFLD), was also observed in a clinical study carried out by Abidov and coworkers [26]. In this study, an increase of energy dissipation, perhaps by thermogenesis, was verified as well in those treated women. Additionally, this xanthophyll carotenoid was also shown to regulate mRNA expression of several cell events and activity of various enzymes [150,153], including lipase activity at the intestinal level [154]. Gammone and D’Orazio [153] and Peng’s group [103] focused on the mechanisms of fucoxanthin and its metabolites as powerful agents against obesity. However in most studies, fucoxanthin was not obtained from marine microalgae, but from brown seaweeds. The main mechanism that explains the anti-obesity properties of fucoxanthin and its metabolites seems to be associated to its capacity to stimulate uncoupling proteins (UCPs) [153,155,156]. UCP-1 (thermogenin), for example, induces fatty acid oxidation and also inhibits oxidative phosphorylation at the mitochondria level, thus enhancing the burning of excess calories, which are dissipated off (energy expenditure) from the body in the form of heat (thermogenesis) [153].

Moreover, different carotenoids, carotenes or xantophylls with different optical (*cis*, *trans*, racemic mixtures) or geometrical (*R*/*S*) forms may present different behaviors at the cell membrane level: they can change the membrane permeability and fluidity, as they can cross along the entire membrane (as is the case for astaxanthin and zeaxanthin) or not (as it is likely for β-carotene and lycopene); this may interfere with their capacity to intercept/react with reactive species/toxins [157]. This could be an explanation for the low ability of β-carotene to inhibit peroxidation of hydrosoluble peroxyradicals, whereas zeaxanthin and astaxanthin are more able to do so [158]. Additionally, various carotenoids may co-exist in animal and human tissues and, therefore, the effect may be synergistic among those lipophilic carotenoids, such as β-carotene and lycopene (acting within the hydrophobic bilayer of cell membranes) and xanthophyll carotenoids that have polar and non-polar regions, such as astaxanthin and zeaxanthin, which can cross the membrane. This explains why a mixture of carotenoids with different chemical characteristics should be administered to prevent oxidative damage and related diseases. Also, the use of the microalgal biomass of a mixture of different genera, such as *Spirulina platensis*, *H. pluvialis* and *Dunaliella salina* could be suggested.

### 4.3. Role of Carotenoids against Reactive Species and Diseases

Liu and co-workers [86] have untangled the link between oxidative stress-generated cell damages in Parkinson’s neurodegenerative disease (PD) and the treatment with the xanthophyll carotenoid astaxanthin. With concentrations as low as 100 nM, astaxanthin demonstrated its AO potential to protect mitochondria of dopaminergic cells, inhibiting peroxidation of lipids, which are severely neurotoxic to the cells, therefore preserving loss of dopaminergic neurons and protecting from PD’s evolution. The protective effect was dose-dependent and associated to the fact that astaxanthin was preferentially accumulated in mitochondria [86]. Furthermore, the oxidative protection of the brain and the neuroprotective effect of astaxanthin may also be due to the fact that this carotenoid can cross the blood-brain barrier as it has been observed in rats’ brain tissue, patented by Tso and Lam in 1996 [159], and in other experimental animals [160]. Astaxanthin also reduced biomarkers 8-OHdG and 4-HNE (Table 3) presented in high levels in cardiac and skeletal muscles of rats, because of the oxidative stress induced by excessive physical exercise [87]. Athletes are particularly affected by oxidative stress due to the high physical efforts, which cause an overproduction of ROS [155]. Thus, an alternative prevention against oxidative stress and the subsequent weakness of the immune system and inflammatory conditions might arise from the intake of carotenoids from marine microalgae, especially astaxanthin and fucoxanthin, two powerful antioxidant carotenoids. In addition, fucoxanthin might be useful, as this marine xanthophyll carotenoid seems to enhance thermogenesis by improving UCPs gene expression and protein activity, decreasing the athlete’s body weight, and therefore improving body performance during sports [155]. In addition to astaxanthin from *H. pluvialis* and fucoxanthin from *O. aurita* and *P*. *tricornutum* [161,162], other carotenoids that can be obtained from various marine microalgae have already been demonstrated to have a wide range of health benefits. This is the case of violaxanthin from *C. ellipsodea* [45,163], canthaxanthin from *C. vulgaris* and *C. striolata var. multistriata* [37,44].

## 5. Why Is There Limited Success of Carotenoids as Anti-Oxidant Agents in Studies/Clinical Trials?

Some researchers have pointed out a wide range of reasons for the failure of carotenoids as AO in tests and clinical trials [3,96,125,164]. The controversial results for the AO properties of carotenoids may possibly be related to an inappropriate choice of the tests for the oxidation products and the biomarkers used, the targeted tissues and the features within the cell. Also the design of the experiment/clinical trial in what concerns the origin and concentration of the right carotenoid, their mechanisms of action and the way of administration, the dose, the duration of the study/therapy, the model/system chosen and the biomarkers selected for the study may have a great influence on the results obtained. Another question has to be asked: are these double-blind placebo-controlled and long-term clinical studies/trials with statistically relevant numbers of individuals? In 2011, Fassett and Coombes [13] referred to the non-existence of such clinical trials, at least for CVD treatment/prevention using astaxanthin.

Following this reasoning, the design of such studies/trials should take into account the chemical and structural characteristics of the various carotenoids, naturally produced *vs.* synthetic, their isomeric and geometric structures, their polarity and redox potential and the pattern of responses elicited in different oxygen pressures in order to choose the most sensitive biomarkers. The probability of the various carotenoids to interact and/or be synergistic with other carotenoids and/or other AO during the metabolic reactions in the whole individual should also be considered. The model should be carefully selected since the human absorption, metabolism and bioavailability of carotenoids is different from other animals, and the chosen animal, as a whole system, implies the existence of different needs, enzymes and responses to different carotenoids. Therefore, the effects at the level of the tissues and/or organs are different from those obtained in chemical tests or even in cell lines, where different responses may be triggered. The best animal models seem to be gerbils, ferrets and calves, as they seem to use and metabolize carotenoids in a similar way as humans [96].

It is worth noting though that when the immune system is deficient, leukocytes cannot produce enough superoxide anion or NO and, thus, a pro-oxidant therapy should be required. Therefore, a carotenoid treatment might be useful in order to trigger this pro-oxidant reaction. The doses administered depend most of the time, if not always, on the chosen carotenoid. Fucoxanthin, for example, has already been demonstrated to exert an AO activity by starting some pro-oxidant effects [128]. Responses obtained also depend on the duration of the study/treatment. Furthermore, β-carotene and other carotenoids can lose their AO properties and act as pro-oxidants when in high concentrations [164,165,166] and in the presence of oxygen [125]. This may also be related with the carotenoid aggregation. Burton and Ingold [125] decribe that, under high oxygen pressures, some carotenoids can suffer auto-oxidation, which may explain the reduction of their AO effectiveness.

Moreover, the AO/pro-oxidant activity of carotenoids strongly depends on the oxygen (O_2_) pressure, and it has to be considered that, except for the lungs and brain, tissues are normally subjected to low O_2_ pressures. Furthermore, β-carotene has demonstrated a better radical scavenging activity under low concentrations of O_2_[125], whilst fucoxanthin is better in anoxic conditions, probably due to the oxygen substituents in the chemical structure [132]; however, in another study, fucoxanthin was less active than α-tocopherol under high oxygen pressures [167].

Additionally, Sangeetha’s group [20,113] demonstrated a higher protection against lipid peroxidation of fucoxanthin when compared to β-carotene, with respect to the increase of the activity of catalase and glutathione (GS) transferase and modulation of Na^+^K^+^-ATPase (Table 4), thus restraining lipid peroxidative stress. These two carotenoids also showed a singlet oxygen quenching ability by inhibiting oxidation of vitamin D [168].

The biochemistry of carotenoids, the way they act, the mechanisms of their interaction with other AO and with different reactive species, the products that result from these reactions and the dual character, either as AO or as pro-oxidants, of carotenoids were summarized by El-Agamey and colleagues [157]. One reason for the pro-oxidant reactions of carotenoids given by these researchers is the depletion of AO, such as vitamins C and E, which may result in the formation of carotenoid radicals that can function as oxidizing agents [157]. They also describe that carotenoids may exert their scavenging activity either by donating electrons or protons, or by forming adducts with the radicals, depending on their lipophilic or hydrophilic nature. Each type of mechanism may depend on the polarity of the carotenoid as well as on the reactivity of the radicals.

The benefits of carotenoids also depend on their molecular structure: carotene carotenoids (β-carotene, lycopene) can only (inter)act within the lipid bilayer of membranes as they do not have polar endings like xanthophyll carotenoids astaxanthin, canthaxanthin and fucoxanthin. This may explain, at least partially, why some carotenoids preserve the membrane integrity better [169] and have protective effects [14], and others may even cause lipid peroxidation and damage in cell membranes [169,170], demonstrating a pro-oxidant potential, when normally administered in high doses [125].

## 6. Conclusions/Final Remarks

Several epidemiologic studies have shown that diets rich in antioxidants reduce the risk of developing several chronic inflammatory diseases, such as Parkinson, diabetes, cancer and CVD. Carotenoids represent the most abundant lipid-soluble phytochemicals, and *in vitro* and *in vivo* studies have suggested that they have a large range of biological properties, such as, antioxidant, anti-inflammatory, modulation of gene expression and many others. 

Microalgae are a potential novel source of bioactive molecules, including a wide range of different carotenoids. Several carotenoids from marine microalgae have been associated with beneficial health effects. These natural compounds have being used as nutraceuticals and food supplements, although the biomass itself may also be incorporated in novel functional food products. By consuming naturally produced carotenoids from microalgae and the biomass in the diets, along with a healthy way of living, with moderate exercise and a decrease of exposure to oxidative agents, humans’ health may improve. This would increase the endogenous antioxidative protection and even prevent some harmful mechanisms and repair damages in the cell and the whole organism. Several pathways involved in the bioactivity of carotenoids have already been disclosed. However, research is still required on the dose-response relations of carotenoids, their metabolites and their effect on the human metabolism.
